# CRISPR/Cas9-Mediated Hitchhike Expression of Functional shRNAs at the Porcine *miR-17-92* Cluster

**DOI:** 10.3390/cells8020113

**Published:** 2019-02-01

**Authors:** Chao Lu, Daxin Pang, Mengjing Li, Hongming Yuan, Tingting Yu, Peixuan Huang, Jianing Li, Xue Chen, Huping Jiao, Zicong Xie, Hongsheng Ouyang

**Affiliations:** Jilin Provincial Key Laboratory of Animal Embryo Engineering, College of Animal Sciences, Jilin University, Changchun 130062, China; swjs092408147@163.com (C.L.); pdx@jlu.edu.cn (D.P.); mjli18@mails.jlu.edu.cn (M.L.); yuanhm17@mails.jlu.edu.cn (H.Y.); ttingyu123@163.com (T.Y.); huangpx16@mails.jlu.edu.cn (P.H.); cool_lijianing@163.com (J.L.); snow1213dawei@163.com (X.C.); jiaohp@jlu.edu.cn (H.J.)

**Keywords:** RNAi, shRNA, miRNA-17-92 cluster, CRISPR/Cas9, classical swine fever virus

## Abstract

Successful RNAi applications depend on strategies allowing stable and persistent expression of minimal gene silencing triggers without perturbing endogenous gene expression. In this study, we proposed an endogenous microRNA (miRNA) cluster as a novel integration site for small hairpin RNAs (shRNAs). We successfully integrated exogenous shRNAs at the porcine *miRNA-17-92* (p*miR-17-92*) cluster via a CRISPR/Cas9-mediated knock-in strategy. The anti-EGFP or anti-CSFV shRNAs could be stably and effectively expressed at the control of the endogenous promoter of the p*miR-17-92* cluster. Importantly, we confirmed that hitchhike expression of anti- classical swine fever (CSFV) shRNA had no effect on cell growth, blastocyst development and endogenous p*miR-17-92* expression in selected transgene (TG) porcine fetal fibroblasts (PFFs) clones. Moreover, these TG PFFs could inhibit the replication of CSFV by half and could be further used for generation of transgenic pigs. Taken together, these results show that our RNA interference (RNAi) expression strategy benefits numerous applications, from miRNA, genome and transgenic research, to gene therapy.

## 1. Introduction

MicroRNAs (miRNAs) are endogenous, small non-coding short RNAs (21~24 nt) that can regulate gene expression mainly through interactions with 3′-untranslated regions of their target genes [1]. The crucial role played by miRNAs in many biological processes, including normal development and cell differentiation/proliferation, is becoming increasingly clear [2]. Approximately 40% of the known human miRNA genes are estimated to form clusters, wherein several mature miRNAs are processed from a single primary transcript. Importantly, clustered miRNAs are transcribed from a specific promoter and have similar expression patterns. As one of the most well-studied miRNA clusters, the *miR-17-92* cluster plays a vital role in normal development [3,4,5]. The *miR-17-92* cluster encodes six distinct miRNAs (*miR-17*, *miR-18a*, *miR-19a*, *miR-19b*, *miR-20a* and *miR-92*) in a single primary transcript compared with common miRNA clusters. Additionally, the *miR-17-92* cluster is highly conserved, and the miRNAs expressed from this cluster are co-regulated, co-expressed, and detectable in most tissues [3,6].

RNAi has recently emerged as a powerful genetic tool for silence gene expression in multiple organisms [7,8]. Previous studies confirmed that ‘‘shRNA-mir’’ cassettes serve as natural substrates in miRNA biogenesis pathways and can trigger potent knockdown, as has been demonstrated for a number of miRNA backbones, including *miR-30*, *miR-155* and *miR-17-92* [9,10,11]. The miR-30-based shRNA design was recommended for its potency and for ease of driving shRNA from a variety of different promoters [12]. Therefore, in this study, we inserted the gene-specific sense and antisense sequences into the miR-30 backbone and synthesized miR-30-based shRNA cassettes. Given that *miR-17-92* can be used for the miRNA backbone, it should be able to drive the expression of shRNAs. Motivated by this idea, we wanted to integrate functional shRNAs at the *miR-17-92* cluster using gene editing technology.

In the past, sequence-specific nuclease technology represented by zinc finger nuclease (ZFN) and transcription activator-like effector nuclease (TALEN) technology has demonstrated its potential in basic research, gene therapy and genetic improvement with its ability to perform fixed-point genome editing with high efficiency [13,14]. However, due to the various technical bottlenecks inherent in ZFN and TALEN, these techniques are not able to achieve rapid development to meet a variety of scientific and clinical needs. Encouragingly, with the advent of the clustered regularly interspaced short palindromic repeats/CRISPR-associated protein 9 (CRISPR/Cas9) system, targeted integration of transgenes by homologous recombination (HR) has been greatly facilitated [15,16,17]. The CRISPR/Cas9 system has been applied to site-specific genome modification in a variety of organisms, including mice [18], pigs [19] and human cells [20,21]. The site-specific integration of functional transgenes is of great value in transgenic animals, gene therapy and biomedical research. Therefore, we expected that the CRISPR/Cas9 technique could achieve functional shRNA insertion at the porcine *miR-17-92* (p*miR-17-92*) cluster, to co-express and further exercise the respective functions of these shRNAs.

Herein, we demonstrated the site-specific integration of functional shRNAs at the p*miR-17-92* cluster using CRISPR/Cas9, and further produced specific shRNA knock-in cells. Precise functional shRNA insertion could be easily introduced and effectively expressed at the endogenous miRNA cluster. Using a comprehensive battery of assays, we confirmed that anti-CSFV shRNA integration and expression had no negative effect on cell proliferation, blastocyst development or endogenous p*miR-17-92* expression in selected TG PFF clones. In particular, an in vitro viral challenge assay demonstrated that these TG PFFs could inhibit the replication of CSFV by half. Our results can provide insight into the potential of the miRNA cluster, and thus will facilitate the use of RNAi technology in cell culture and vertebrate animals.

## 2. Materials and Methods

### 2.1. Ethics Statement

All animal studies were approved by the Animal Welfare and Research Ethics Committee at Jilin University (Approval ID: 201706002), and all procedures were conducted strictly in accordance with the Guide for the Care and Use of Laboratory Animals. All surgeries were performed under anesthesia, and every effort was made to minimize animal suffering.

### 2.2. Plasmid Construction

Three single-guide RNAs (sgRNAs) targeting the p*miR-17-92* cluster were designed using online software (http://crispr.mit.edu/). The sgRNA oligonucleotides were annealed and ligated to the linearized pX330 vector (Addgene no. 42230) using the method described by Zhang at the Broad Institute of MIT.

The miR-30-based shRNA donor vector contained a 0.5 kb left homologous arm (HA), shRNA and a 0.8 kb right HA. The HAs were amplified from the porcine genome, and the shRNA sequences were synthesized by Suzhou Genema (Suzhou, China). The resulting fragments were cloned into the pLB-simple vector using an EasyGeno Assembly Cloning kit (TIANGEN, Beijing, China).

### 2.3. EGFP Reporter Cell Lines

The sgRNA91/Cas9 (Addgene no. 42230) and PUC57-pRosa26-EGFP donor vector have been described previously and preserved well in our laboratory [22]. SgRNA91/Cas9 was introduced into the PK-15 cell line with the PUC57-pRosa26-EGFP donor by electroporation. Positive *EGFP* knock in cell colonies were obtained by the limiting dilution method and fluorescence microscopy. After three days of culture, the cells were trypsinized and plated at 2000–3000 cells per 10 cm dish to enable the generation of single-cell colonies. Before picking the single-cell colonies, the surrounding heterocells had to be scraped off to ensure the purity of cell colonies. After approximately 8 days of culture, single-cell colonies were picked and propagated for further analysis. To confirm the cell clones for the site-specific knock-in, we assessed the 5′-junction and 3′-junction by PCR. The primers and sequences are shown in Supplementary Table S1. The PCR products spanning the target sites in the clones were sequenced directly. The identified positive cell clones were observed at 72 h post-electroporation using fluorescence microscopy (Olympus BX51, Tokyo, Japan) and further analyzed using a BD Accuri C6 flow cytometer (BD Biosciences, New York, NY, USA).

### 2.4. Cell Culture and Transfection

Primary cultures of PFFs were prepared as described previously [23]. Primary PFFs were isolated from 33-day-old fetuses and cultured in a medium consisting of Dulbecco’s Modified Eagle’s Medium (DMEM, Gibco, Carlsbad, CA, USA) supplemented with 15% fetal bovine serum (FBS) and 1% penicillin/streptomycin. PK-15 and *EGFP* knock-in PK-15 (EGFP-KI-PK) cell lines were cultured in DMEM (Gibco) containing 5% FBS (Gibco) and 1% penicillin/streptomycin. The medium was changed every 2 days. All cells were cultured in a humidified incubator at 37 °C with 5% CO_2_.

For cell transfection, approximately 3 × 10^6^ cells and 30–60 μg of the corresponding plasmids (30 μg of pX330, 30 μg of pX330 plus 30 μg of donor vector) were suspended in 300 μL of Opti-MEM (Gibco, Grand Island, New York, USA) in 2 mm gap cuvettes, and electroporated by using the specified parameters with a BTX-ECM 2001. The electroporation parameters for those cell lines are listed in Supplementary Table S2.

### 2.5. Selection of siRNAs

All siRNAs were designed and synthesized by Suzhou Genema (Suzhou, China). The siRNAs were respectively introduced into EGFP-KI-PK or PK-15 cells by electroporation at a suitable concentration. These siRNA-transfected cells were seeded into 24-well plates with three replicates for each siRNA. For other siRNAs targeting *EGFP*, the siRNA-transfected cells were observed 48 h post-electroporation using fluorescence microscopy (Olympus BX51, Tokyo, Japan) under appropriate excitation filters. The harvested cells were washed twice, resuspended in 500 μL of PBS and analyzed using a BD Accuri C6 flow cytometer (BD Biosciences, New York, NY, USA). In addition, siRNA 2-1 targeting CSFV in our laboratory has been validated as an effective siRNA by indirect immunoinfluscent assay (IFA) and can be used in subsequent experiments without screening [24].

### 2.6. Selection of Cell Clones

At 48 h post-transfection, the cells were inoculated into ten 100 mm dishes at an appropriate density (2000–3000 cells/dish). Individual cell clones were picked and cultured into 24-well plates. After reaching 80% or more confluence, the cell clones were subcultured, and 20% of each cell clone was lysed with 10 μL NP40 lysis buffer (0.45% NP40 plus 0.6% proteinase K) for 1 h at 56 °C and 10 min at 95 °C. The lysate was used as a PCR template and was subjected to 1% agarose gel electrophoresis. Additionally, the PCR products were sequenced to confirm the knock-in events. The primers were designed to cover the target sequence and the sequences are shown in Supplementary Table S1. To confirm the cell clones for the site-specific knock-in, some PCR products were selected for ligation into a pLB vector (Tiangen, Beijing, China) and sequenced to determine the exact sequences. The selected positive cell clones were used to test the blastocyst formation rate as well as developmental ability by somatic cell nuclear transfer (SCNT), as described previously [25].

### 2.7. IFA

The proliferation of positive shRNA knock-in clones was determined by an IFA. These positive shRNA knock-in clones were inoculated with CSFV at 5 h post-transfection. After 72 h incubation at 37 °C, PFFs were washed three times with cold PBS, and fixed overnight in 80% pre-cooled acetone. The fixed cells were incubated with an anti-CSFV polyclonal antibody (PAb) (1:100) for 2 h at 37 °C, washed three times with cold PBS, and then incubated with a fluorescein isothiocyanate (FITC) labelled goat anti-pig IgG (1:100) antibody (Sigma-Aldrich, New York, NY, USA) for 1 h at 37 °C. After three washes with PBS, the cells were examined under a fluorescence microscope (Olympus BX51, Tokyo, Japan).

### 2.8. Quantitative Real Time PCR Analysis

Total RNA was extracted using TRIzol-A+ reagent (Tiangen, Beijing, China). First-strand cDNAs were generated through reverse transcription using total RNA and oligo-dT primers. Porcine *GAPDH* served as the reference gene for the relative expression of *EGFP*.

Small RNAs and miRNAs were isolated by using a miRcute miRNA Isolation Kit (Tiangen, Beijing, China). From purified RNA, complementary DNA was synthesized using a miRcute miRNA First-Strand cDNA Synthesis Kit (Tiangen, Beijing, China). Quantitative real time PCR (qPCR) was also performed using a miRcute miRNA qPCR Detection Kit (Tiangen, Beijing, China) according to the manufacturer′s instructions. The shRNA expression was normalized to the expression of endogenous *U6* using the 2^−ΔΔCt^ method. 

qPCR was performed to examine CSFV in cells. Viral genomic RNA was isolated by using TRIzol-A+ (Tiangen, Beijing, China) according to the manufacturer’s instructions. A standard curve was generated to detect the viral load in each sample by 10-fold serial dilutions of viral lysates ranging from 10^8^ to 10^2^, and Ct values and the copy numbers of viruses were assessed. These primer sequences are listed in Supplementary Table S3.

### 2.9. Cell Proliferation Assay

The proliferation assay for TG PFFs was performed using a Cell Counting Kit-8 (DOJINDO, Kumamoto, Japan), according to the manufacturer’s recommendations. In order to determine the optimal incubation time, cell activity of the transgenic cells was detected by incubation with CCK-8 for a certain time to measure the absorbance at 450 nm. Briefly, TG PFFs were seeded in 96-well plates (1 × 10^4^ per well) and incubated in fresh DMEM medium for 24 h at 37 °C with 5% CO_2_. After pre incubation, the plates were further incubated in a humidified incubator for a suitable period of time. After the cells were washed with PBS, 100 μL of DMEM medium containing 10% WST-8 solution was added to the wells, and the plate was incubated for 1.5 h. Then, the absorbance of each well was measured at 450 nm by a microplate reader (Infinite 200 PRO, TECAN, Zürich, Switzerland). 

### 2.10. SCNT and Blastocyst Formation

The shRNA knock-in positive PFF clones cultured in 24-well plates were used to perform SCNT, which was carried out according to a previously described method [25]. Positive PFF clones were injected into enucleated oocytes to form reconstructed embryos. The reconstructed embryos were subsequently activated and cultured as described previously [26]. Some embryos were cultured for 6–7 days to test the blastocyst formation rate and developmental ability. Donor cells were divided into wild-type (WT) and shRNA-knock-in (TG) groups for somatic cell nuclear transfer, and the effects of shRNA insertion on early embryo development were evaluated by comparing the differences in blastocyst development rates between the two groups.

### 2.11. Off-Target Analysis

All potential off-target sites (OTSs) were predicted by scanning the porcine genome using BLAST and analyzed via PCR and DNA sequencing to determine off-target effects. All predicted OTSs are shown in corresponding Figure and the primer sequences are listed in Supplementary Table S4. Briefly, a T7 endonuclease I (T7E1) assay was performed according to the manufacturer’s protocol. The PCR products surrounding OTSs were purified and digested with T7E1 for 30 min at 37 °C. All digested products were then analyzed by electrophoresis in 2% agarose gels.

### 2.12. Statistical Analysis

The data were statistically analyzed using GraphPad Prism v5.0 (GraphPad Software Inc., San Diego, CA, USA) (*t*-test). All data are presented as the mean ± S.E.M. Values were considered statistically significant at *p* < 0.05. The results are representative of more than three individual experiments.

## 3. Results

### 3.1. Genomic Organization and Expression of the Porcine miR-17-92 Cluster

**Figure 1:**
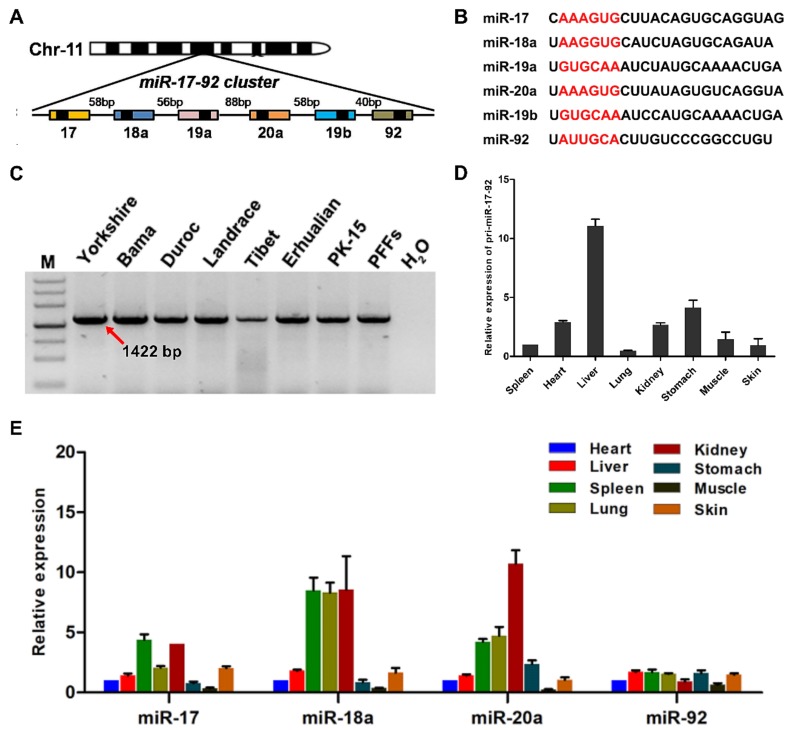
Genomic organization and expression of the p*miR-17-92* cluster. (**A**) Schematic representation of the p*miR-17-92* cluster. The p*miR-17-92* cluster is located in an intergenic region on chromosome 11 in the porcine genome. Pre-miRNAs are indicated as color-coded boxes. Black boxes correspond to the mature miRNA. (**B**) Sequence comparison of the miRNAs expressed from the p*miR-17-92* cluster. The seed sequences are indicated in red. (**C**) Polymorphism analysis of the p*miR-17-92* cluster in different pig breeds and in two different cell lines. M: DNA marker III. PFFs: porcine fetal fibroblasts from Yorkshire (China) pigs. (**D**) Relative expression of *pri-miR-17-92* in different adult porcine tissues as determined by real-time PCR. All values are the mean ± S.E.M., n = 3. **(E)** Relative expression of miRNAs expressed from the p*miR-17-92* cluster in a variety of tissues as determined by qPCR. All values are the mean ± S.E.M., n = 3.

As a polycistronic miRNA gene [27], the porcine *miR-17-92* (p*miR-17-92*) cluster encodes six miRNAs (*miR-17*, *miR-18a*, *miR-19a*, *miR-20a*, *miR-19b*, and *miR-92*), which are tightly grouped within a 782 base-pair region of pig genome chromosome 11 (Figure 1A,B). The conservation of the *miR-17-92* cluster was analyzed by PCR with primer C1/C2 (Figure 1C) and further confirmed by Sanger sequencing. Sequence alignments showed high conservation in six different porcine breeds as well as in two different cell lines (Supplementary Figure S1). qPCR assays showed that the *pri-miR-17-92* was widely expressed in all porcine tissues tested. Among all the analyzed tissues, the expression of *pri-miR-17-92* in the liver was highest, and the expression of *pri-miR-17-92* in the lung was lowest (Figure 1D). In addition, our results revealed that the examined miRNAs expressed from the *miR-17-92* cluster showed varying degrees of expression in these tested tissues (Figure 1E). In particular, the expression levels of *miR-18a* and *miR-20a* in the kidney were higher than those in other tissues tested (Figure 1E). These results indicated that the porcine *miR-17-92* gene cluster was ubiquitously expressed in a wide variety of tissues.

### 3.2. Designation of the sgRNA for the pmiR-17-92 Cluster

**Figure 2:**
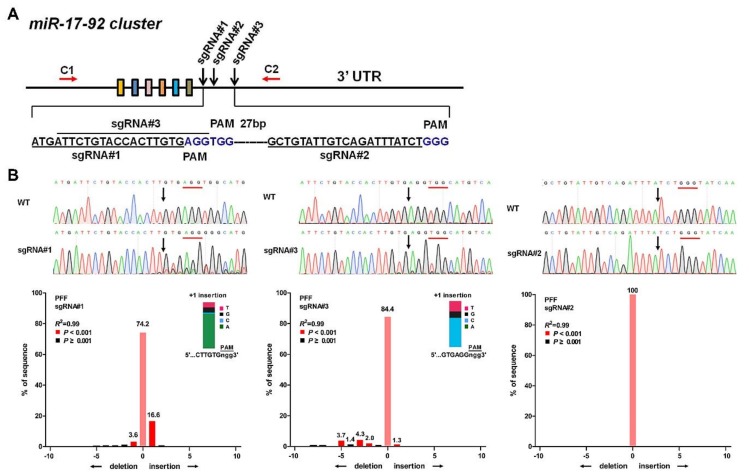
TIDE analysis of p*miR-17-92* targeted with designed sgRNAs in PFFs. (**A**) Schematic representation of sgRNAs specific to the 3′UTR of p*miR-17-92*. The sgRNA sequence is highlighted in black, and PAM is highlighted in blue. (**B**) A pool of PFF cells treated with Cas9 alone (control) and cells treated with Cas9 and a p*miR-17-92*-targeting sgRNA (sgRNA#1, sgRNA#2 or sgRNA#3) (sample) were analyzed by TIDE. Insets: Prediction of the inserted base for +1 insertions. PAMs are underlined in red and the cleavage sites are labelled with black arrows.

Given this broad expression pattern in porcine adult tissues and two different porcine cell lines, we were then interested in determining whether this locus could be targeted through a CRISPR/Cas9-based knock-in strategy. Because its promoter region and internal structure are complicated, we first designed three guide RNAs (sgRNAs) to target the 3’-untranslated region (3’UTR) of the p*miR-17-92* cluster (Figure 2A). To assess the targeting efficiency of sgRNAs, three specific sgRNA/Cas9 encoding vectors were transfected into PFFs via electroporation. After three days of culture, genomic DNA was isolated from these cells, and PCR products covering the target site were examined through T7 endonuclease I (T7E1) assays and Sanger sequencing. Obvious multi-peaks around the Cas9 cleavage site were observed in the chromatogram, and TIDE analysis [28,29] showed that the gene editing efficiency of sgRNA#1 (24.3%) was higher than that of sgRNA#3 and sgRNA#2 (Figure 2B). These results suggested that the mutations had been introduced and that sgRNA#1 achieved a higher targeting efficiency than the other two sgRNAs. T7E1 assays also showed that sgRNA#1 presented the desired targeting efficiency (Supplementary Figure S2A). To further determine the mutation efficiency, the PCR amplicons of sgRNA#1 were TA cloned and further sequenced to assess the non-homologous end-joining (NHEJ) events (Supplementary Figure S2B). Together, these results indicated that sgRNA#1 could be used to effectively target the p*miR-17-92* cluster in subsequent experiments.

### 3.3. CRISPR/Cas9 Mediated Site-Specific Anti-EGFP shRNA Insertion at pmiRNA-17-92 in PK-15-EGFP-KI Cells

**Figure 3:**
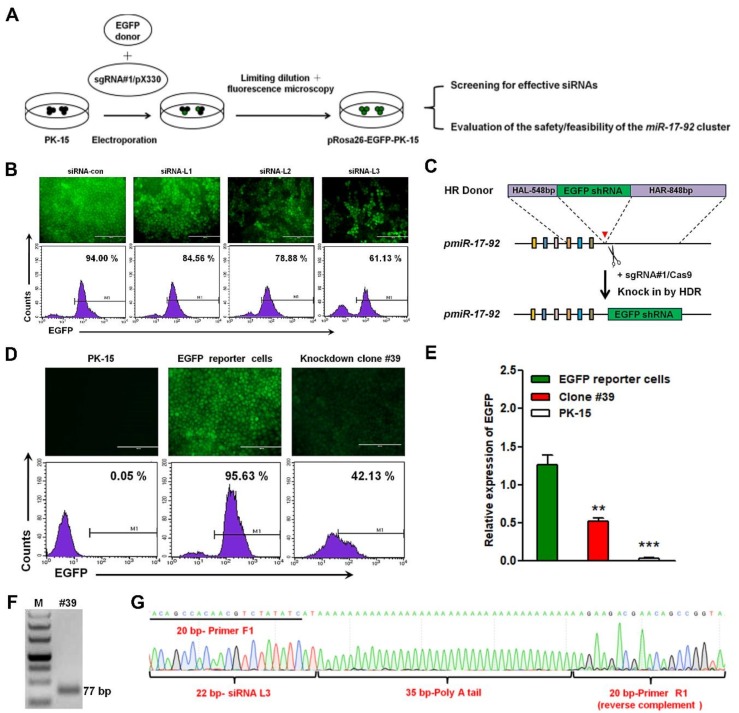
Construction and application of EGFP reporter cell lines via a CRISPR/Cas9 system. (**A**) Schematic representation of the generation of pRosa26-EGFP-PK-15 reporter cell lines. (**B**) SiRNAs (siRNA-L1~L3) were individually transfected into EGFP reporter cell lines by electroporation. EGFP fluorescence was analyzed via fluorescence microscopy and FACS at 72 h post-transfection. (**C**) Schematic representation of the anti-EGFP shRNA knock-in strategy into the p*miR-17-92* cluster. (**D**) EGFP fluorescence was analyzed via fluorescence microscopy and FACS at 72 h post-transfection. (**E**) Relative expression of *EGFP* in EGFP reporter cells and positive clone #39. Con: EGFP reporter cells. All values are the mean ± S.E.M., n = 3. ** *p* < 0.01, *** *p* < 0.001. (**F**) Expression of siRNA-L3 in positive clone #39 was confirmed by RT-PCR. M: 50 bp DNA ladder. The size of the target amplicon was 77 bp. (**G**) Sanger sequencing analyses were used to further confirm the expression of siRNA-L3 in positive clone #39. The target amplicon with primer 1F/1R corresponds to 3F.

Recently, our previous study reported a CRISPR/Cas9 mediated site-specific *EGFP* knock-in reporter in PFFs at the p*Rosa26* locus [22]. In the current study, we first utilized the same strategy to produce *EGFP* knock-in reporter PK-15 cells (EGFP-KI-PK) at the p*Rosa26* locus, which can be used to effectively evaluate expression activities of the shRNA site-specific knock-in at the p*miR-17-92* locus (Figure 3A). Fluorescence microscopy and FACS results indicated that the EGFP-KI-PK reporter cell line was successfully constructed (Figure 3B,D). The site-specific *EGFP* insertion was confirmed by PCR with specific primers and the sequencing results are shown in Supplementary Figure S3. Next, our goal was to select siRNA-EGFP that could effectively inhibit the expression of the *EGFP* gene. Three siRNAs were designed (Table 1) and respectively introduced into EGFP-KI-PK cells by electroporation. At 72 h post-transfection, the knockdown efficiency of these siRNAs was observed by fluorescence microscopy and further analyzed by FACS. Among the three tested siRNAs, siRNA-L3 showed the highest knockdown efficiency (Figure 3B). 

To determine whether p*miR-17-92* can drive exogenous shRNA expression, a targeting HR donor vector without a promoter or selectable marker was constructed (Supplementary Figure S4). The anti-EGFP shRNA donor vector was introduced into EGFP-KI-PK cells along with the sgRNA#1/Cas9 vector by electroporation (Figure 3C). The anti-EGFP shRNA knock-in clones were selected by the limiting dilution method and the fluorescence microscopy. Among the selected clones, site-specific shRNA insertions were confirmed by PCR and Sanger sequencing (Supplementary Figure S5). Then, these identified positive knockdown clones were further analyzed by FACS, and the relative expression of the *EGFP* gene in these positive clones was examined by quantitative PCR. In particular, the intensity of green fluorescent cells in clone #39 decreased to 42.13% (Figure 3D) and the relative expression of the *EGFP* gene also decreased correspondingly (Figure 3E). Finally, the expression of siRNA-L3 was detected by RT-PCR with primers F1/R1 and further confirmed by sequencing (Figure 3F,G). As indicated above, siRNA-L3 was effectively transcribed and expressed under the control of the endogenous p*miR-17-92* promoter, and the expression of shRNA-EGFP could effectively inhibit the expression of the *EGFP* gene in EGFP-KI-PK reporter cell line. Altogether, these results demonstrated that the p*miR-17-92* locus could be used for site-specific knock-in and effective expression of functional shRNA. It suggested that the p*miR-17-92* cluster may have potential for the preparation of transgenic PFFs and even transgenic animals.

### 3.4. CRISPR/Cas9 Mediated Site-Specific Anti-CSFV shRNA Insertion in Porcine Fetal Fibroblasts

**Figure 4:**
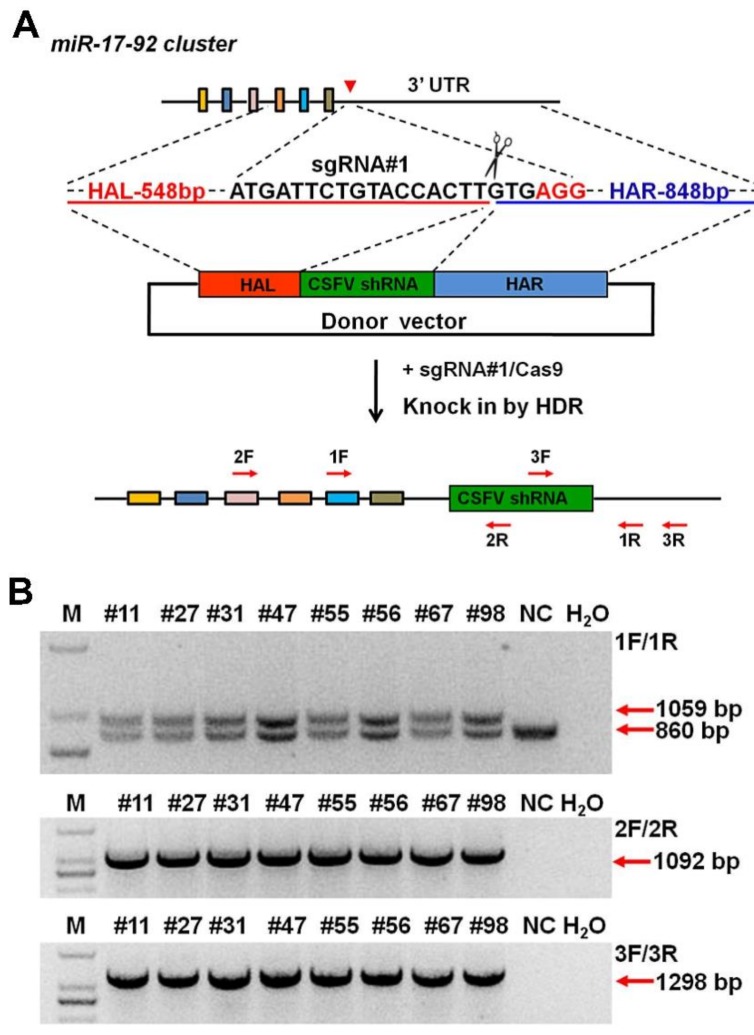
Selection of anti-CSFV shRNA knock-in cell clones. (**A**) Strategy of anti-CSFV shRNA knock-in into the p*miR-17-92* cluster. (**B**) PCR analysis of positive PFF clones using specific primer. The 1F/1R primers were used to determine homozygosity or heterozygosity, the 2F/2R primers amplified the 5′ junction, and the 3F/3R primers amplified the 3′ junction. A hybrid band was formed by knock-in band (1059 bp) and wild-type band (860 bp) in the process of genomic PCR with 1F/1R; The corresponding sizes of the PCR amplicons with 2F/2R and 3F/3R are 1092 and 1298 bp. M: DNA Marker 2000. WT: negative control. H_2_O: blank control.

**Figure 5:**
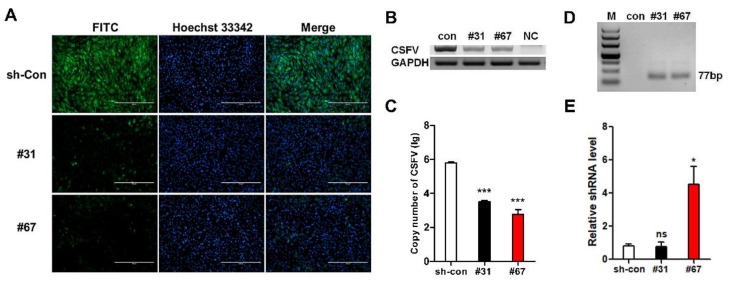
Verification of the antiviral ability of TG PFF clones. (**A**) Virus resistance in identified positive PFF clones #31 and #67 was examined by IFA. sh-Con: scrambled shRNA transgenic PFFs. (**B**) Antiviral ability in positive PFF clones #31 and #67 was further assessed by RT-PCR at 72 h post-infection. (**C**) qPCR results showed the copy number of CSFV virus in positive PFF clones #31 and #67. The values displayed on the Y axis are calculated in lg. All values are the mean ± S.E.M., n = 3. *** *p* < 0.001. (**D**) Expression of the target siRNA in corresponding positive PFFs clones #31 and #67 was further confirmed by RT-PCR. M: 50 bp DNA ladder. (**E**) Relative expression level of anti-CSFV shRNA in positive PFF clones #31 and #67 were confirmed by qPCR. All values are the mean ± S.E.M., n = 3. ns, not significant. * *p* < 0.05.

**Figure 6:**
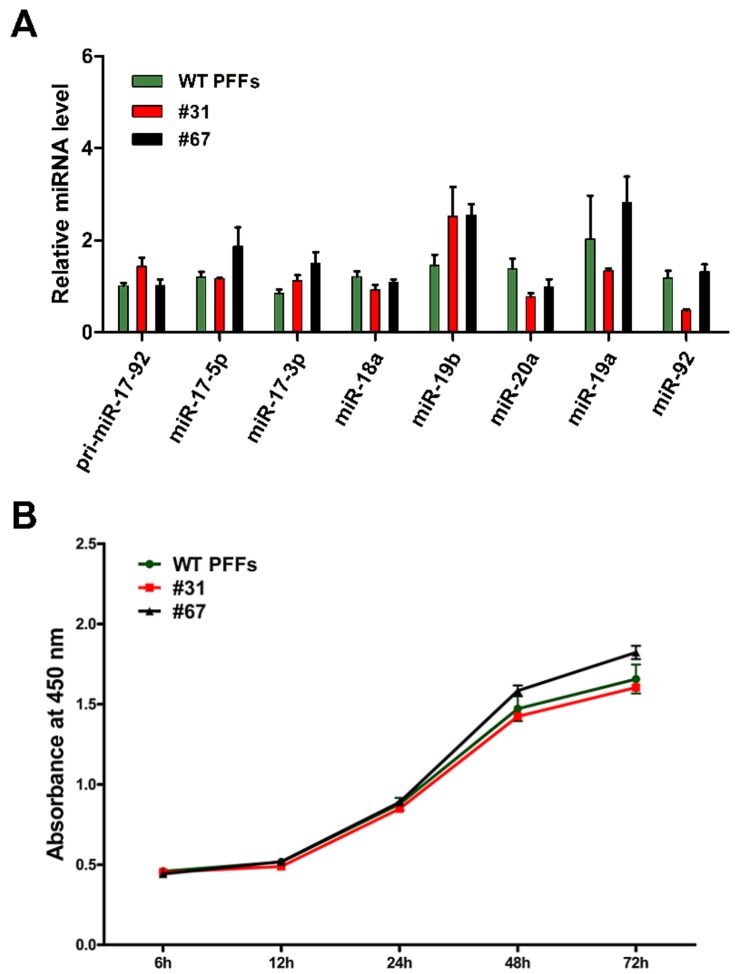
qPCR detection and Cell proliferation assay in TG PFF clones. (**A**) Relative expression levels of miRNAs expressed from the p*miR-17-92* cluster in positive PFF clones #31 and #67 were confirmed by qPCR. All values are the mean ± S.E.M., n = 3. No significant difference was found among the groups. (**B**) The effect of the anti-CSFV shRNA insertion on cell proliferation in positive PFF clones. All values are the mean ± S.E.M., n = 3. No significant difference was found among the groups.

As described above, site-specific anti-CSFV shRNA knock-in was introduced into PFFs. The HR donor was constructed using the same homology arm as the pLB-EGFP-shRNA vector (Supplementary Figure S6). The anti-CSFV shRNA knock-in strategy is shown in Figure 4A. Single-cell clones were also obtained by limiting dilution, and eight positive clones were identified from 96 monoclonal cells by PCR and Sanger sequencing (Figure 4B and Figure S7). Then, an in vitro viral challenge assay was performed, and the inhibition efficiency of these positive clones was determined by IFA [30,31,32]. The IFA results showed that these positive clones significantly inhibited the proliferation of CSFV compared with the control cells (Figure 5A,B). In addition, we detected the relative expression of shRNA and the copy number difference in CSFV in positive clones #31 and #67 (Figure 5C). Finally, the expression of antiviral shRNA was detected by RT-PCR (Figure 5D,E) and further confirmed by sequencing (Supplementary Figure S8). These results confirmed that the knock-in of anti-CSFV shRNA in PFFs at the p*miR-17-92* locus could enhance resistance to CSFV infection. Next, we also examined the relative expression level of the *miR-17-92* cluster between wild-type (WT) PFFs and positive clones (#31 and #67). Our results showed that the miRNAs expressed from the p*miR-17-92* cluster in positive clones were all expressed normally, and that there was no significant difference in expression level compared with WT PFFs (Figure 6A). To assess the adverse effects caused by the shRNA knock-in, the cell proliferation assay for TG PFFs was performed. The CCK-8 results indicated that the cell proliferation of positive clones #31 and #67 was basically consistent with that of WT PFFs (Figure 6B and Figure S9). Furthermore, these positive knock-in PFFs were used as the donor cells for SCNT to evaluate the blastocyst development rate [25]. Encouragingly, our results demonstrated that WT PFFs (WT group) and the positive clones (TG group) shared a similar blastocyst development rate (19.18 ± 0.6527% vs. 19.16 ± 0.2074%, *p* > 0.05, n = 3) (Table 2). Hence, these results demonstrated that the site-specific anti-CSFV shRNA insertion had no significant adverse effects on cell growth or blastocyst development (Supplementary Figure S10). This finding strongly suggested that these knock-in PFFs could be further used to generate anti-CSFV TG pigs by SCNT.

### 3.5. Off-Target Analysis

**Figure 7:**
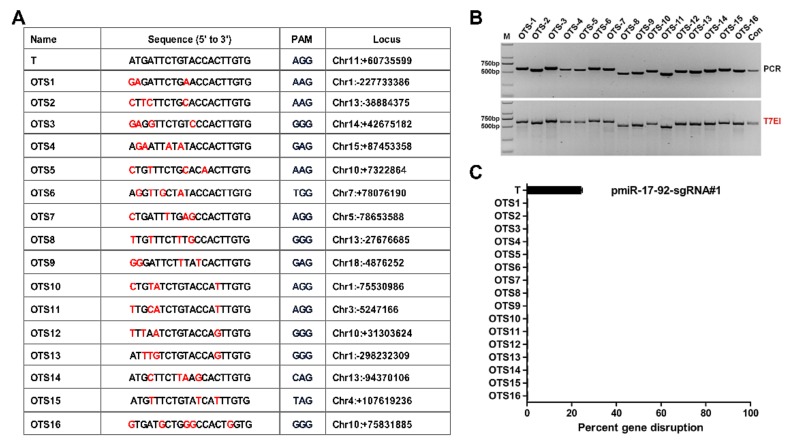
Off-target site analysis in positive PFF clones. (**A**) List of potential off-target sites homologous to sgRNA#1/cas9. T represents target site and OTS1~OTS16 represents 16 potential off-target sites. PAM sequences are labelled in blue. Base substitutions are shown in red. (**B**) PCR products covering 16 potential off-target sites were subjected to the T7E1 assay and no mutation was found in any potential off-target sites. M: DNA Marker 2000. (**C**) Off-target mutagenesis measurement of p*miR-17-92* cluster. Indel frequencies were measured by TIDE analysis. All TIDE analyses below the detection sensitivity of 1.5% were set to 0%. All values are the mean ± S.E.M., n = 3. T: target site. OTS: off-target site.

Despite significant advances in the CRISPR/Cas9 system, concerns remain over the potential for off-target effects [33,34]. According to the prediction of CRISPR design, a total of 16 OTSs was selected and are listed in Figure 7A. The mixed DNA of selected anti-CSFV positive PFF clones was evaluated by a T7E1 assay (Figure 7B) and further confirmed by Sanger sequencing (Supplementary Figure S11) using primer pairs (Supplementary Table S4). Consistent with the above results, the TIDE analysis [35] showed that no off-target mutations were observed in the 16 potential OTSs when targeting p*miR-17-92* with Cas9 (Figure 7C).

## 4. Discussion

Sequence-specific gene silencing by short hairpin RNAs (shRNAs) has recently emerged as an indispensable tool for understanding gene function and a promising avenue for gene therapy. On the basis of early studies, the advantages of miR-30-based shRNA cassettes are simplicity, availability and compatibility with a number of standard vector systems. In general, 100–200 bases of the primary miRNA-30 transcript are included in the shRNA cassette, which facilitates the processing of shRNAs [36]. 

However, the insufficient delivery of shRNA is the major obstacle that limits its application and efficiency. The development of the gene editing technology, especially the CRISPR/Cas9 system, provides us with an alternative way to overcome these problems. With its high efficiency and simplicity of manipulation, CRISPR/Cas9 technology has achieved numerous successes in various species as a powerful genome engineering tool. Many measures have been taken to improve the low knock-in efficiency of CRISPR/Cas9, including positive and negative selection methods. However, introducing drug-selectable marker genes into cells and animals may interfere with internal gene expression, and deleting a selectable marker is a laborious process. Therefore, in this study, we chemically synthesized gene-specific miR-30-based shRNA cassettes according to the design principles, and no selectable markers or promoters were introduced in our designed targeting donor vectors. We first inserted effective anti-EGFP shRNA at the p*miR-17-92* locus into EGFP-KI-PK reporter cells to evaluate its safety/feasibility, inducing the knockdown of the *EGFP* gene, and indicating that the integration and efficient expression of anti-EGFP shRNA could be achieved at this locus. These results confirmed that the endogenous p*miR-17-92* promoter could be utilized to drive the expression of exogenous shRNA genes. This result gives us confidence in integrating more different functional shRNAs into the endogenous miRNA cluster to target different mRNAs and virus via CRISPR/Cas9. To verify these important assumptions, further in-depth and systematic research (e.g., the structures and sequences of the miRNA cluster) will be performed in the following studies.

CSFV infection results in highly significant economic losses in the swine industry [37,38]. With the rampant prevalence of CSF, increasing attention has been paid to the prevention and control of CSF. Currently, CSF is controlled by a non-vaccination, stamping-out policy or prophylactic vaccination. Many measures have been taken, but the control effects are sometimes unsatisfactory. RNAi is an evolutionarily conserved mechanism of gene regulation that uses small RNAs produced to guide suppression of complementary transcripts. RNAi-mediated targeting of viral RNAs has been clearly demonstrated in plants, invertebrates and mammalians cells as an antiviral defense mechanism [39] and therefore, this technique holds huge promise for combatting viral infection [40].

To facilitate the control and eradication of CSF, we expected to effectively resist to CSFV infection via CRISPR/Cas9-based knock-in strategy. In this study, we also demonstrated that anti-CSFV shRNA knock-in was introduced into PFFs. By combining CRISPR/Cas9 with the limited dilution method, we acquired marker-free anti-CSFV shRNA knock-in PFFs. These positive knock-in PFFs we identified had the following characteristics: (i) carried a single targeted anti-CSFV shRNA integration, (ii) showed insignificant changes in endogenous *miRNA-17-92* expression, (iii) stably expressed anti-CSFV shRNA, (iv) efficiently and specifically inhibited the replication of CSFV and (v) had little effect on cell proliferation and blastocyst development, together illustrating the great potential of our RNAi expression strategy.

CRISPR/Cas9 has accelerated the pace of the development in biology, agriculture, engineering, and biomedicine as a powerful genetic editing tool. However, the potential for off-target effect limits its application [41,42]. In this study, we selected 16 potential OTSs for the target site by T7E1 assay and further confirmed by Sanger sequencing, and found that all potential off-target sites were unaffected in these TG PFFs. However, a comprehensive analytical approach such as whole-genome sequencing is still needed for future detection of off-target effects of the CRISPR/cas9 system because off-target mutations may occur at other possible sites beyond those predicted sites. Additionally, recently, researchers have developed a variety of new Cas9 variants (eSpCas9, Hypa Cas9, HeFSpCas9s, etc.) with higher specificity that can effectively reduce off-target effects [43,44,45]. 

In summary, we demonstrated an endogenous miRNA cluster for stable, precise and potent expression of exogenous functional shRNAs using CRISPR/Cas9 technology and RNAi technology. Importantly, exogenous functional shRNAs could be readily inserted, and efficiently expressed, and the integration of these shRNAs had no effect on the expression of genes in the endogenous p*miR-17-92* cluster. Furthermore, the anti-CSFV TG PFFs mentioned above may be further used for the generation of transgenic pigs. Our results provide insight into characterizing the potential of miRNA or miRNA clusters for shRNA hitchhike expression, which may facilitate the development of transgenic research, RNAi technology and gene therapy.

## Figures and Tables

**Table 1:** The target genes, names and corresponding sequences of siRNAs.

Target Gene	Name of siRNA	Sense (5’–3’)	Antisense (5’–3’)
*EGFP*	siRNA-L1 siRNA-L2 siRNA-L3	UGUGAUCGCGCUUCUCGUUGGGTT UGUUGUAGUUGUACUCCAGCUUTT AUGAUAUAGACGUUGUGGCUGUTT	CCCAACGAGAAGCGCGAUCACATT AAGCUGGAGUACAACUACAACATT ACAGCCACAACGUCUAUAUCAUTT
*CSFV*	siRNA-2-1	UCCUGUACAUUCAACUACGCAATT	UUGCGUAGUUGAAUGUACAGGATT
Control	siRNA-Con	UUCUCCGAACGUGUCACGUAACTT	GUUACGUGACACGUUCGGAGAATT

**Table 2:** Summary of blastocyst development in the WT and TG groups.

Group	Donor Cells	Donor Cells Number	Blastocyst Number	Blastocyst Rate ^1^
WT	wild-type PFFs-#1	156	29	18.59%
	wild-type PFFs-#2	166	34	20.48%
	wild-type PFFs-#3	130	24	18.46%
TG	shRNA-KI-clone-#31	160	30	18.75%
	shRNA-KI-clone-#67	139	27	19.42%
	shRNA-KI-clone-#98	145	28	19.31%

^1^ Blastocyst rate (%) = (Blastocyst number/Donor Cells number) × 100%.

## Supplementary Materials

The following are available online at http://www.mdpi.com/2073-4409/8/2/113/s1, Figure S1: Sequences alignment of the *miRNA-17-92* cluster from different pig breeds and different cell lines, FigureS2: T7E1 assay of sgRNA#1 at the p*miRNA-17-92* locus in PFFs, Figure S3: Results of genomic PCR analysis confirmed the *EGFP* knock-in events at the p*ROSA26* locus, Figure S4: Composition and structure of the pLB-EGFP shRNA-KI donor vector, Figure S5: Results of genomic PCR analysis confirmed the anti-EGFP shRNA knock-in events at the p*miR-17-92* cluster, Figure S6: Composition and structure of the pLB-CSFV shRNA-KI donor vector, Figure S7: Sanger sequencing results of the PCR products obtained with specific primer pairs, Figure S8: Sanger sequencing analyses of the expression of the target CSFV siRNA 2-1 in identified positive knock-in PFF clones, Figure S9: Effect on the cellular activity of different PFFs under different incubation times with CCK-8, Figure S10: The reconstructed embryos were cultured in vitro for approximately 6 days until the blastocyst stage, Figure S11: Sanger sequencing analyses of PCR amplicons that spanning the potential off-target sites, Table S1: Primers and corresponding sequences for knock-in events, Table S2: Electroporation parameters for various cell lines, Table S3: Primers and corresponding sequences for real-time PCR analysis, Table S4: Primers for PCR amplification of the off-target sites.
