# Supplementary material for: CRISPR/Cas9-Mediated Hitchhike Expression of Functional shRNAs at the Porcine miR-17-92 Cluster
Source: Cells. 2019 Feb 1;8(2):113. doi: 10.3390/cells8020113 (PMC6406430; doi:10.3390/cells8020113)
Supplement: Supplementary file 1 [file cells-08-00113-s001.pdf]

Supplementary Files

CRISPR/Cas9-mediated hitchhike expression of functional shRNAs at the porcine miR-17-92 cluster

Chao Lu[1], Daxin Pang[1], Mengjing Li, Hongming Yuan, Tingting Yu, Peixuan Huang, Jianing LI, Xue Chen, Huping Jiao, Zicong Xie*, Hongsheng Ouyang*

Jilin Provincial Key Laboratory of Animal Embryo Engineering, College of Animal Sciences, Jilin University, Changchun, Jilin Province, No.5333 Xi'an Road, Changchun 130062, Jilin Province, People's Republic of China.

[1] These authors contributed equally to this work.

* Correspondence: Zicong Xie & Hongsheng Ouyang

E-mail: wx11196@163.com(Z.X.); ouyh@jlu.edu.cn(H.O.).

Tel: (86)431-87835175; Fax: (86) 431-87980131

Supplementary Figures

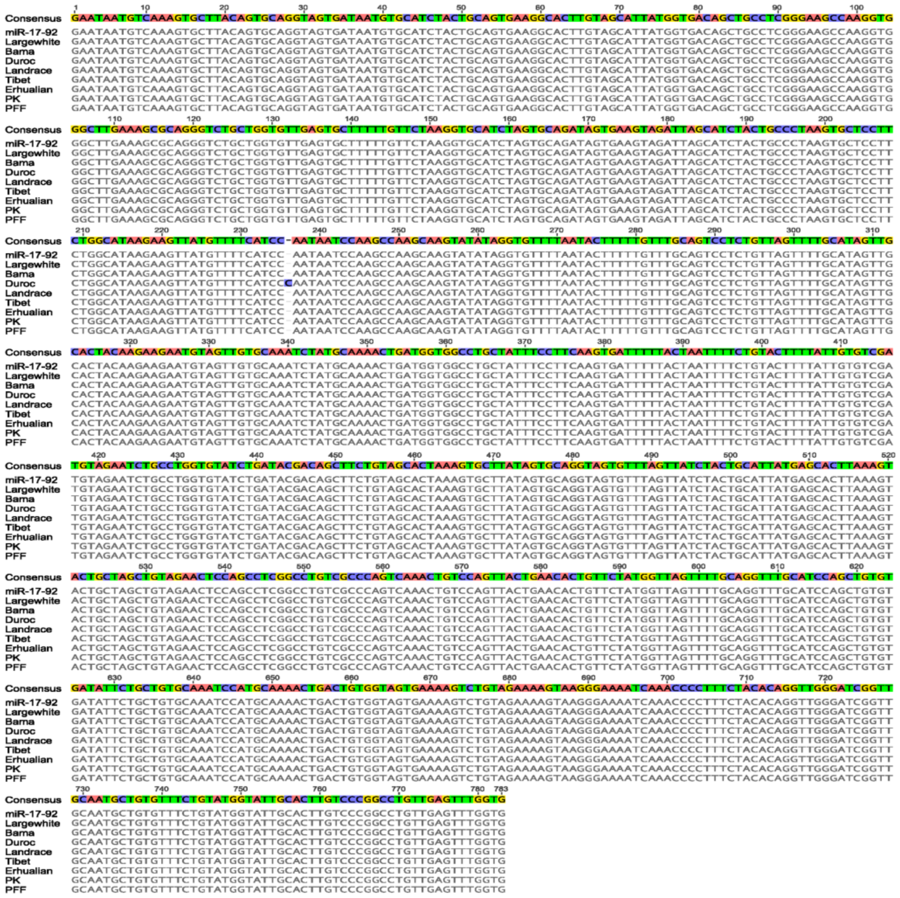

Figure S1 Sequence alignment of the miRNA-17-92 clusters from different pig breeds and different cell lines.

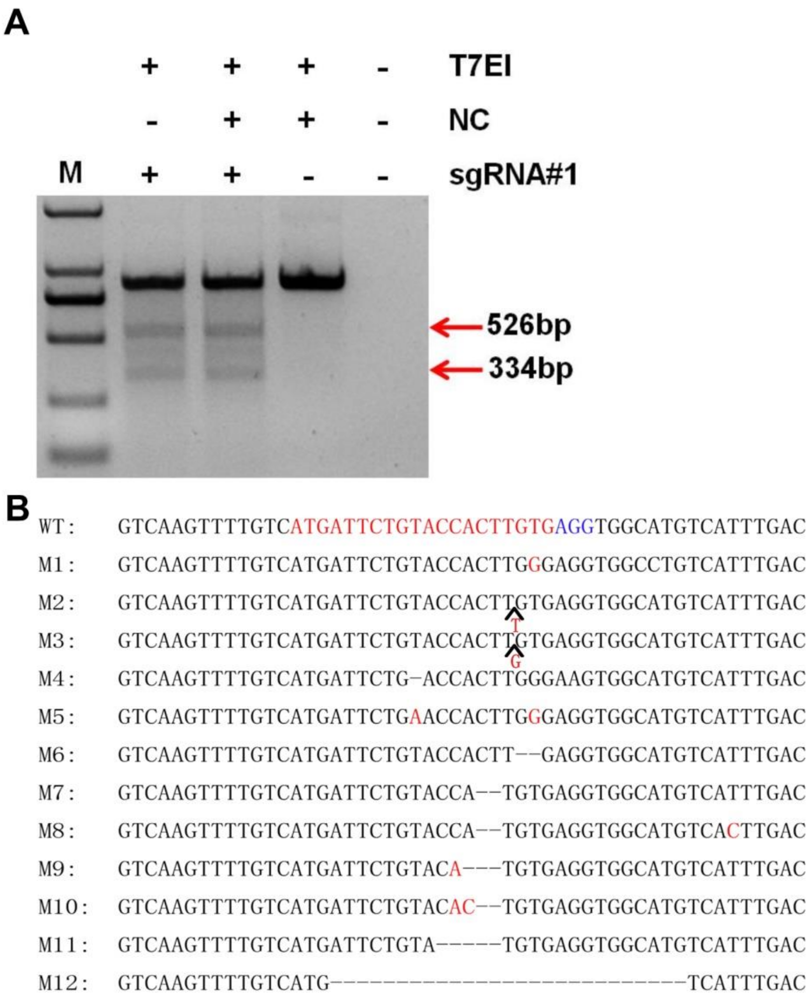

Figure S2 T7E1 assay of sgRNA#1 at the pmiRNA-17-92 locus in PFFs. (A) The mutation efficiency for each sgRNA#1/Cas9 was determined by using the T7E1 cleavage assay. M: DNA Marker 2000. The red arrows indicated cleaved amplicons (526bp and 334bp). (B) The TA cloning and Sanger sequencing were analysed for deletions and insertions (indels). The wild-type sequence is located on the first line (WT), and the mutated sequences from the TA cloning are arranged below (M1~M12). The target site is highlighted in red; the PAM is indicated in blue.

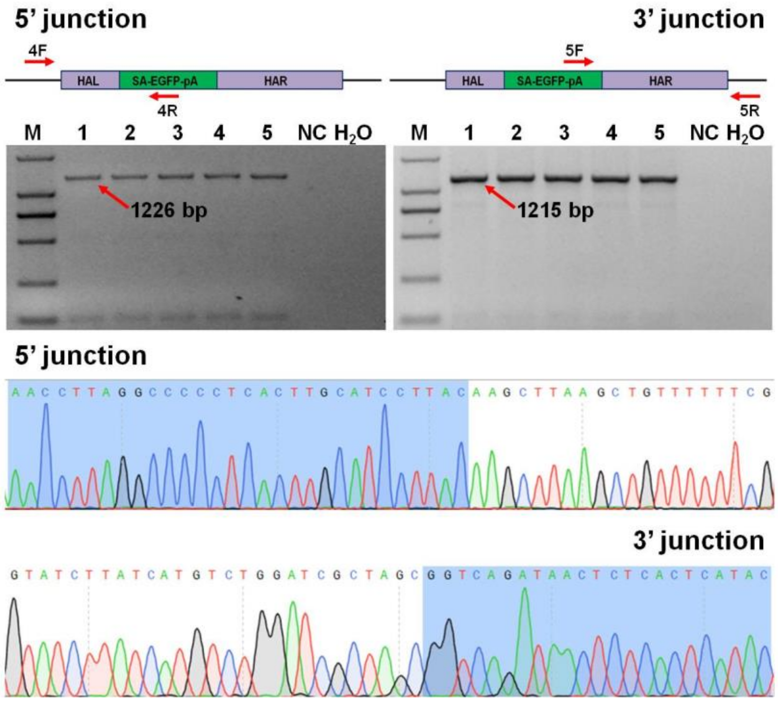

Figure S3 Results of genomic PCR analysis confirmed the *EGFP* knock-in events at the *pROSA26* locus. The 4F/4R primers amplified the 5′ junction, and the 5F/5R primers amplified the 3′ junction. The sequences of these primers are listed in Supplementary Table S1. Lanes 1-5 represent the *EGFP* knock-in-positive cell clones. NC: negative control; M: DNA Marker 2000.

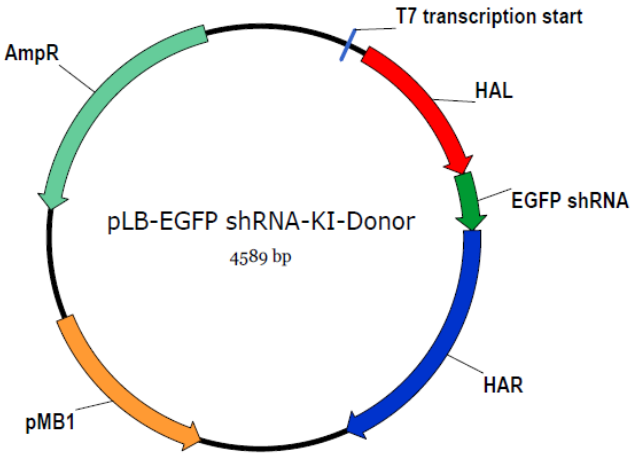

Figure S4 Composition and structure of the pLB-EGFP shRNA-KI donor vector.

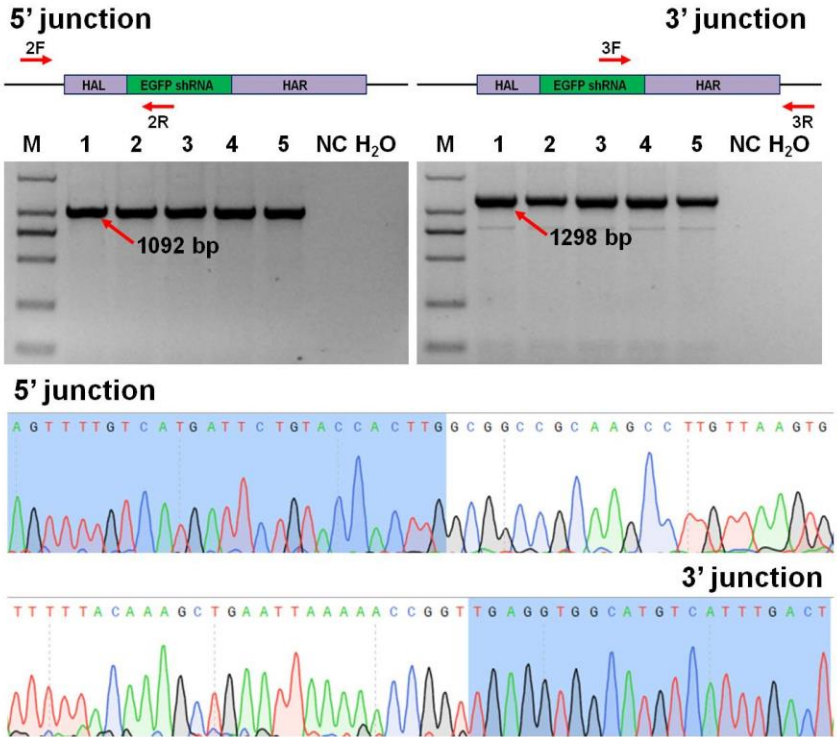

Figure S5 Results of genomic PCR analysis confirmed the anti-EGFP shRNA knock-in events at the pmiR-17-92 cluster. The 2F/2R primers amplified the 5′ junction, and the 3F/3R primers amplified the 3′ junction. The sequences of these primers are listed in Supplementary Table S1. Lanes 1-5 represent the anti-EGFP shRNA knock-in positive cell clones. NC: negative control; M: DNA Marker 2000.

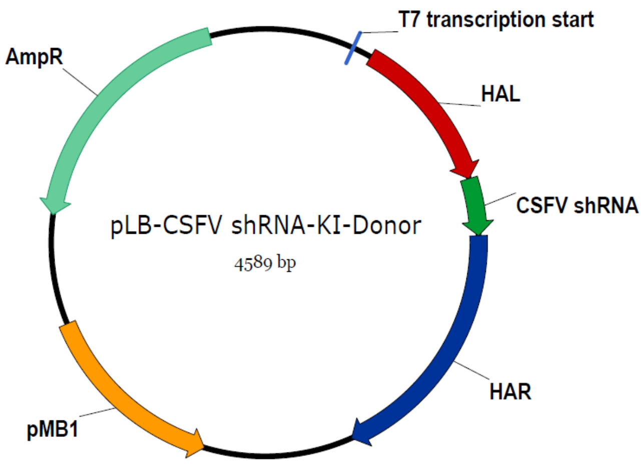

Figure S6 Composition and structure of the pLB-CSFV shRNA-KI donor vector.

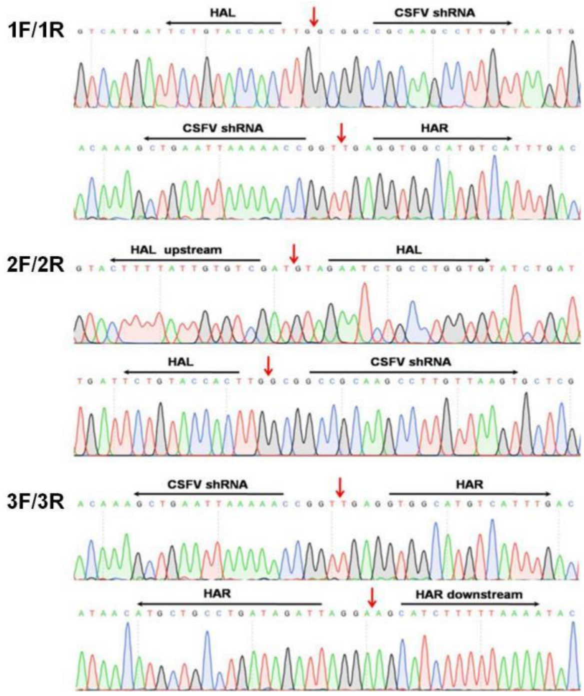

Figure S7 Sanger sequencing results of the PCR products obtained with specific primer pairs (1F/1R, 2F/2R or 3F/3R).

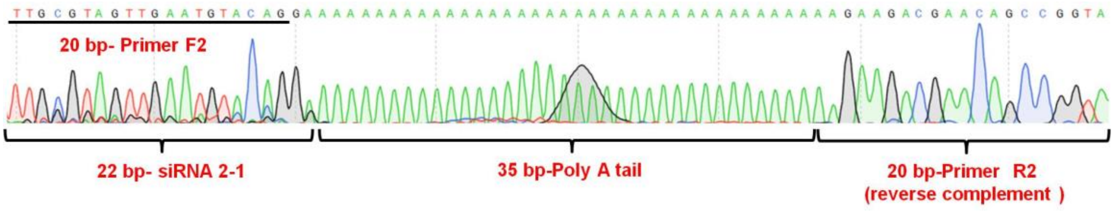

Figure S8 Sanger sequencing analyses of the expression of the target CSFV siRNA 2-1 in identified positive knock-in PFF clones**.**

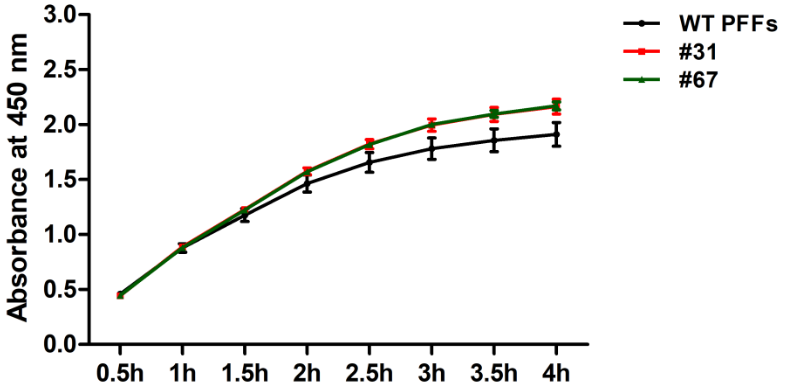

Figure S9 Effect on the cellular activity of different PFFs under different incubation times with CCK-8. Cells were seeded at a density of 1×10^4 per well in 96-well plates and incubated in fresh complete medium for 24 h. All values are the mean ± S.E.M. (n=3). No significant difference was found among the groups.

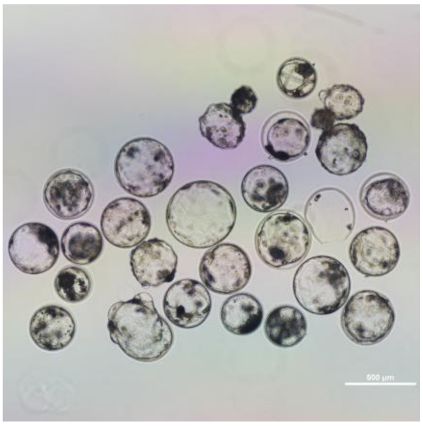

Figure S10 The reconstructed embryos were cultured in vitro for approximately 6 days until the blastocyst stage. Positive knock-in PFFs were mixed and used as donor cells to perform SCNT and examine the developmental potency of reconstructed embryos.

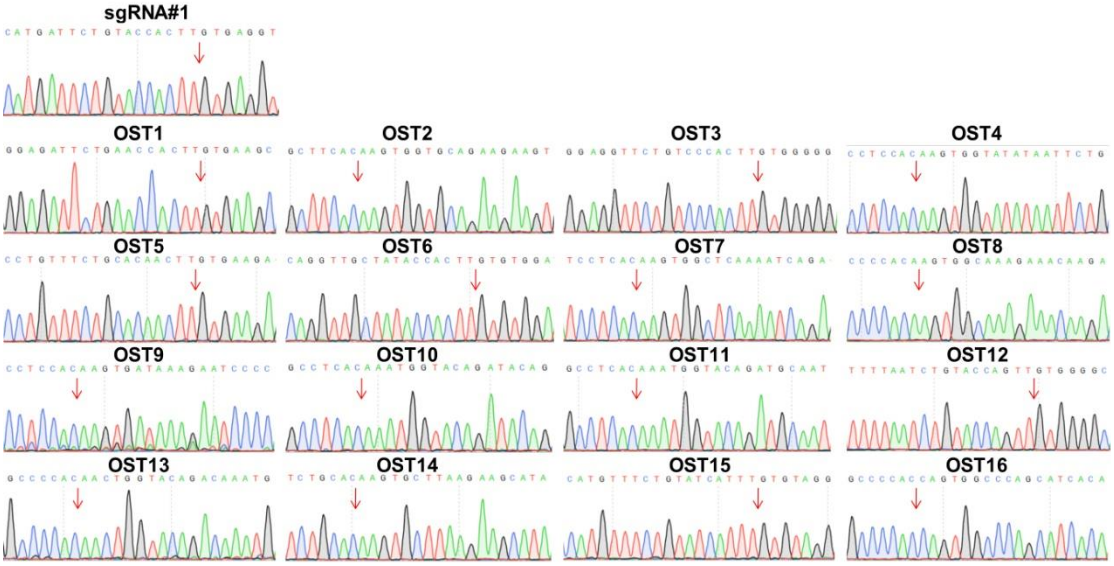

Figure S11 Sanger sequencing analyses of PCR amplicons that spanning the potential off-target sites. The potential cleavage sites are labeled with red arrow.

Supplementary Tables

Table S1 Primers and corresponding sequences for knock-in events

Primer Name	Sequence (5′-3′)
C1	AAGTATGCATTTGGGAGTGGC
C2	GCAAAAGGCATATCATCTCCG
1F	ATTCTGCTGTGCAAATCCATG
1R	CCGCTCAACTCCAATACTCTT
2F	GCAGGAATAAAGAGACCATCAC
2R	CGCTCACTGTCAACAGCAATAT
3F	AGTGTCTGCCTACTGCCTCGGA
3R	GTATGGCTTGGTAGGTGTAAAC
4F	GGTCCCAAATGAGCGAAAC
4R	AGCGAGCACTTAACAAGGC
5F	GATACATTTTTACAAAGCTGAATTA
5R	CACTACCAAACATACAAAAGAACTA

Table S2 Electroporation parameters for various cell lines (BTX-ECM2001)

Cell line	Set voltage (V)	Pulse length (ms)	Number of pulses
PFF	340	1	3
PK-15	300	1	3
EGFP-KI-PK	300	1	3

Table S3 Primers and corresponding sequences for real-time PCR analysis

Primer Name	Sequence (5′-3′)
GAPDH-F	GCCATCACCATCTTCCAGG
GAPDH-R	TCACGCCCATCACAAACAT
EGFP-F	GCGCACCATCTTCTTCAA
EGFP-R	GCTTGTCGGCCATGATATAG
U6-F	CTCGCTTCGGCAGCACA
U6-R	AACGCTTCACGAATTTGCGT
ssc-pri-F	ATCTACTGCCCTAAGTGCTCCTTC
ssc-pri-R	ACTATGCAAAACTAACAGAGGACTGC
ssc-miR-17-5p	CAAAGTGCTTACAGTGCAGGTA
ssc-miR-17-3p	ACTGCAGTGAAGGCACTTGTAG
ssc-miR-18a	TAAGGTGCATCTAGTGCAGATA
ssc-miR-19a	TGTGCAAATCTATGCAAAACTGA
ssc-miR-20a	TAAAGTGCTTATAGTGCAGGTA
ssc-miR-19b	TGTGCAAATCTATGCAAAACTGA
ssc-miR-92	TATTGCACTTGTCCCGGCCTGT

Table S4 Primers for PCR amplification of the off-target sites

Primer	Sequence (5' To 3')	Amplicon size (bp)
OTS1	CTGTCTAATCACACCTGTCCA<br>CCCTTTGATAATTCCAGCCCA	599
OTS2	TGGTTTCTGGCATGTGTTCA<br>GCACTGAGAGGCCATCAAAA	556
OTS3	GGAACAGTTGATCTTTGCAG<br>GACAATCATTTGAGTGCCTG	546
OTS4	CTACACCACAGCTCAGGGCA<br>TGACATTTGGAGGTGAGCGA	612
OTS5	AGCAGCCATTCACCAATCAG<br>GATTTGATCCCCTAGCCTGG	575
OTS6	CTGAAGGGGAGATAGGGTTG<br>CTGGCACAGCAGAAACGAAT	561
OTS7	ATAGCAAGCAGGAGGGAGGT<br>AGAAGCCACACCGATCTCAT	603
OTS8	AGACATTTAGTGCTCTCCCAAC<br>ATTTCCATCCAACCTCACTCTT	587
OTS9	ACTAAAACTTACCCACCAGG<br>CAGAAGATGGGAAGAGTGTG	484
OTS10	TCTCATTGTTGCCGTCCATC<br>CCTTGAGGGAGCAGTGTGGT	501
OTS11	AGGCTGAGCATTTTGTAACC<br>CACTGTGTTTTCTATTCGCA	553
OTS12	CATTGTCAACCACACCTCA<br>CTCGCATCCCTTCTCTCAG	489
OTS13	CTGCTAGGAAGGGGAGAATC<br>GGTGGCATTTTGAAGAACTG	571
OTS14	GTCTCCTCTCCCATTCTCTC<br>TGTTGTCTAAACCAAGCGAG	565
OTS15	AGCACAAGAGATGGCTCACT<br>CTGCCCAACTGCTCTCCTAT	601
OTS16	AGGCTGGACGGACAAGAGGA<br>TAAGCACTTTGGGCTACATA	619
